# Phylogeography of Aegean green toads (*Bufo viridis* subgroup): continental hybrid swarm *vs*. insular diversification with discovery of a new island endemic

**DOI:** 10.1186/s12862-018-1179-0

**Published:** 2018-05-02

**Authors:** Christophe Dufresnes, Petros Lymberakis, Panagiotis Kornilios, Romain Savary, Nicolas Perrin, Matthias Stöck

**Affiliations:** 1 Department of Ecology & Evolution, University of Lausanne, Biophore Building, 1015 Lausanne, Switzerland; 2 Department of Animal and Plant Sciences, University of Sheffield, Alfred Denny Building, Western Bank, Sheffield, S10 2TN UK; 3 Natural History Museum of Crete, University of Crete, Knosos Av, P.O. Box 2208, 71409 Irakleio, Crete Greece; 4 Section of Animal Biology, Department of Biology, School of Natural Sciences, University of Patras, GR-26500 Patras, Greece; 5 Department of Biology, University of Washington, Box 351800, Seattle, WA 98195-1800 USA; 6 Leibniz-Institute of Freshwater Ecology and Inland Fisheries (IGB), Müggelseedamm 301, D-12587 Berlin, Germany

## Abstract

**Background:**

Debated aspects in speciation research concern the amount of gene flow between incipient species under secondary contact and the modes by which post-zygotic isolation accumulates. Secondary contact zones of allopatric lineages, involving varying levels of divergence, provide natural settings for comparative studies, for which the Aegean (Eastern Mediterranean) geography offers unique scenarios. In Palearctic green toads (*Bufo viridis* subgroup or *Bufotes*), Plio-Pleistocene (~ 2.6 Mya) diverged species show a sharp transition without contemporary gene flow, while younger lineages, diverged in the Lower-Pleistocene (~ 1.9 Mya), admix over tens of kilometers. Here, we conducted a fine-scale multilocus phylogeographic analysis of continental and insular green toads from the Aegean, where a third pair of taxa, involving Mid-Pleistocene diverged (~ 1.5 Mya) mitochondrial lineages, earlier tentatively named *viridis* and *variabilis*, (co-)occurs.

**Results:**

We discovered a new lineage, endemic to Naxos (Central Cyclades), while coastal islands and Crete feature weak genetic differentiation from the continent. In continental Greece, both lineages, *viridis* and *variabilis*, form a hybrid swarm, involving massive mitochondrial and nuclear admixture over hundreds of kilometers, without obvious selection against hybrids.

**Conclusions:**

The genetic signatures of insular Aegean toads appear governed by bathymetry and Quaternary sea level changes, resulting in long-term isolation (Central Cyclades: Naxos) and recent land-bridges (coastal islands). Conversely, Crete has been isolated since the end of the Messinian salinity crisis (5.3 My) and Cretan populations thus likely result from human-mediated colonization, at least since Antiquity, from Peloponnese and Anatolia. Comparisons of green toad hybrid zones support the idea that post-zygotic hybrid incompatibilities accumulate gradually over the genome. In this radiation, only one million years of divergence separate a scenario of complete reproductive isolation, from a secondary contact resulting in near panmixia.

**Electronic supplementary material:**

The online version of this article (10.1186/s12862-018-1179-0) contains supplementary material, which is available to authorized users.

## Background

A debated aspect in speciation research is the mode by which reproductive isolation between diverging lineages evolves under natural conditions [1]. On the one hand, reproductive isolation may build up gradually during the time in allopatry, as multiple, weak genetic incompatibilities accumulate over the genome. On the other hand, reproductive isolation may evolve in a stepwise manner, resulting from few key “speciation genes” that underlie traits, responsible for pre- or post-zygotic isolation (“magic traits”, [2]), as e.g. involved in ecological adaptation. In the case of allopatric speciation, one way to disentangle these hypotheses is to test whether hybridizability in secondary contact zones negatively correlates with the divergence time of lineages from a single radiation. Such a relationship is suggested for a few systems, such as fishes [3, 4], amphibians [5–9], reptiles [10] and mammals [11]. This supports a progressive buildup of “barriers restricting gene flow locally in the genome (that) lead to patterns of heterogeneity” [1] and thus reproductive isolation can evolve in allopatry. Yet, the time taken to speciate seems to vary between radiations, possibly even among related taxonomic groups (e.g. anuran amphibians, [6, 7]), but comprehensive evidence from additional models is still missing. Comparative hybrid zone analyses can shed light on the controversial role of gene flow in driving speciation [1]. While gene flow can promote reinforcement [12, 13] if the diverging genomes have become sufficiently incompatible for hybrids to be selected against, it may rather bring the two incipient species back into panmixia, if their genomes are still compatible. These aspects also have crucial implications for conservation. The timeframe of reproductive isolation may serve as a baseline to hierarchize diverging lineages into taxonomic categories, and especially validate the status of cryptic, incipient species, upon which the (administrative) priority for their protection might depend.

In particular, the northern circum-Mediterranean regions with the Iberian, Italian and Balkan Peninsulas, are the “theaters” of the initial and final steps of vicariant speciation processes for many terrestrial and freshwater lineages. While often providing the Plio-Pleistocene source populations and glacial refugia during Quaternary climatic fluctuations, their geographic and environmental complexity also initiated and directed genetic divergence in many organisms [14]. Especially, the Aegean (Eastern Mediterranean) geography offers unique phylogeographic scenarios. Its topographic features, combined with changes in sea levels according to glacial cycles, which consecutively connected and isolated island populations, strongly promoted diversity at the regional level. As diverging lineages eventually came into secondary contact and experienced gene flow, they either merged, thus “reversing” speciation, or evolved reproductive barriers (possibly by reinforcement), so as to “seal” this allopatric process. These features make several Mediterranean regions, and specifically the Aegean, a natural experimental arena for evolutionary and conservation biologists to study the mechanisms underlying the formation and maintenance of intra- and inter-specific diversity [15].

With at least twelve mitochondrial haplotype groups, the Western Palearctic radiation of green toads (*Bufo viridis* subgroup, *Bufotes*) constitutes a fascinating system to address such questions [16–18]. This group, with the oldest living lineages found in the Western Himalayas [16], further diversified in the central and eastern Mediterranean regions, forming allo- or parapatric lineages in North Africa (*B. boulengeri*), Sicily (*B. siculus*), on the Apennine Peninsula and Western Mediterranean islands (*B. balearicus*), the Balkan Peninsula (*B. viridis*) and in Anatolia (*B. variabilis*). The northern parts of Central and Eastern Europe were post-glacially colonized by two mitochondrial lineages, namely *B. viridis* (Laurenti, 1768) and *B. variabilis* (Pallas, 1769). The latter name was “tentatively referred to” by Stöck et al. [16], and its unclarified taxonomic status was discussed [19]. As widespread ectothermic vertebrates, green toads are showing significant phylogeographic responses to Quaternary geoclimatic oscillations, and are thus expected to be highly informative on the biogeographic processes leading to diverging, and eventually speciating lineages. Green toads may serve as a natural model to understand how reproductive isolation evolves over time in anuran amphibians. Studying secondary contact zones, previous work showed that the Plio-Pleistocene-diverged *B. siculus* and *B. balearicus* (divergence 2.6 My) developed advanced reproductive isolation with scare if any recent gene flow [18], while *B. balearicus* exhibited asymmetric introgression with its Pleistocene counterpart *B. viridis* (divergence 1.9 My) across their hybrid zone in the Po Plain of Northern Italy [6].

Here, we focus on green toads from the Eastern-Mediterranean region, specifically across the Aegean Sea. This area is one of Europe’s richest biodiversity hotspots, including a well-documented colonization history of the herpetofauna from three major source regions [20]. The phylogeography of the Aegean has been imprinted by the periods between initiation (12 Mya) and ending (9 Mya) of the Mid Aegean Trench formation, the Messinian Salinity Crisis (ending at 5.3 Mya, [21]), the climate oscillations during the Pleistocene and anthropogenic influences on the ecosystems, including voluntary and involuntary animal introductions during the Holocene [20, 22]. This multifaceted history of the Aegean region includes myriads of islands, featuring complex patterns of species assemblages, regional diversification and insular (neo-)endemism (notably on the Cyclades), involving at least six distinct biogeographic regions [23]. Combined with the diverse influences from Anatolian and European (as well as African) biota, several mechanisms have been proposed to promote the Aegean diversity, like habitat heterogeneity including mountainous regions on large islands driving ecological isolation, as well as frequent over-sea dispersal, potentially mediated by antic human civilizations since 10′000 years ago [20]. Geologically, major islands date back to the end of the Messinian (5.3 Mya; [21]) and thus were already in place during the Plio-Pleistocene, when paleo-water levels started to greatly fluctuate throughout the Quaternary. During the Wurm glaciation (in the north: the Weichselian) some 18,000 years ago (Last Glacial Maximum, LGM; [24]), the sea levels were accurately recorded at 121 ± 5 m lower than today. Thus, coastal islands were frequently connected by land-bridges and at last only were disconnected in the Holocene, while remote islands remained completely disconnected, potentially since the Messinian (e.g. Crete, Karpathos, Rhodes, Naxos). In this way, the bathymetry of the Aegean has played a key role for the distribution of genetic diversity in terrestrial species, at least since the Plio-Pleistocene [20, 25]. Yet, few studies have been conducted to study this role at the level of very closely related terrestrial vertebrates. Since green toads are present over most parts of the Archipelago, they present an excellent system to test whether intraspecific divergence mirrors the history of isolation and connections of many islands and to elucidate their biogeographic patterns.

Second, the mainland, especially of northeastern Greece and neighboring Turkey, adjacent to the Aegean Sea, constitutes a suture zone between Anatolian and Balkan lineages [7, 15, 26, 27]. This is also the case in green toads, where the mitochondrial sister taxa *viridis* and *variabilis* came into secondary contact in continental Greece [16, 17]. Since these two lineages originated after the split with *B. siculus* and *B. balearicus*, an analysis of their hybrid zone provides a valuable third point to examine the relationship between hybridizability and divergence time and thus speciation in this anuran radiation.

To better understand the terrestrial Aegean phylogeography of closely related vertebrates as well as to elucidate the genetic interactions of the presumably young lineages *viridis* and *variabilis* on the mitochondrial and nuclear levels, we conducted a fine-scale multilocus phylogeography of European green toads across their Aegean range. Specifically, (1) we tested whether any endemic lineages are found on Aegean islands and if lineage distributions and divergences can be attributed to past sea level changes. (2) We documented patterns of introgression between the continental lineages of *viridis* and *variabilis*, and compared them to other green toad hybrid zones [6, 18], in order to infer the timeframe of reproductive isolation under natural conditions.

## Methods

### Sampling and DNA extraction

A total of 757 individuals from 131 localities were included in this study, densely covering continental Greece and neighboring countries, the Izmir region in coastal Turkey, and multiple populations from the Greek Aegean islands of Chios, Crete, Ikaria, Kythera, Lemnos, Lesvos, Naxos, Rhodes, Thasos and Serifos (Additional file 1: Table S1). Among these samples, 76 originated from collections of the Natural History Museum of Crete (NHMC). The remaining samples were collected during the breeding periods (February–June) from 2011 to 2013, and consisted of non-invasive buccal swabs (wild caught adults) and ethanol-preserved tissues (larvae, museum specimens). DNA was extracted using the Qiagen Biosprint Robotic Workstation.

### Mitochondrial and nuclear genotyping and sequencing

First, we amplified and sequenced ~ 900 bp of the mitochondrial control (*D-loop*) region in 165 individuals, representative of most populations. PCR amplification was carried out in 25 μl reaction volumes, containing DNA template (~ 2–3 μl), Qiagen Buffer (1×), dNTPs (0.19 mM), primers *CtrlB-H* and *Cytb-AL* ([28]; 0.5 μM each; cf. [16]) and Qiagen Taq (0.625 units), performed under the following conditions: an initial cycle of 2′ at 96 °C, 45″ at 52 °C and 2′ at 72 °C; 38 cycles of 30″ at 94 °C, 45″ at 52 °C and 1′30″ at 72 °C; 5′ at 72 °C.

For all remaining 687 green toads, we mito-typed PCR amplicons of the control region, using a novel enzyme-restriction procedure, designed with the NEBcutter 2.0 tool (New England Biolabs), which allowed to differentiate mitotypes between these two species. This procedure included: (1) PCR amplification as above; (2) enzymatic restriction by *NlaIII* (New England Biolabs) for 1 h at 37 °C in 9 μL reaction volumes, containing PCR amplicons (3 μL), NEB4 buffer (0.7×), BSA (0.1×) and *NlaIII* (1 unit); (3) migration of digested products on a 2% agarose gel for 1 h 30 min at 100 V; and (4) scoring of restriction patterns on the gel: the digested PCR products of *viridis* featured three bands of ca. 190, 320 and 390 bp, while those of *variabilis* featured three bands of 120, 330 and 450 bp respectively.

Second, in order to assess nuclear phylogenetic relationships among the lineages occurring in the study area, we amplified and cloned (TOPO-TA cloning kit, Invitrogen; methods: [6, 17]) the nuclear *α-tropomyosin* intron 5 (*tropo*, ~ 530 bp) in a representative subset of individuals (*n* = 16), chosen based on geography and initial sequencing results. A minimum of eight clones per individual was sequenced. Cloning was required to fully resolve sequence alleles despite the presence of indel polymorphisms in this intron.

Third, we genotyped eight microsatellite loci, cross-amplifying and polymorphic in both lineages (*viridis*, *variabilis*), successfully obtained from 635 individuals from 110 localities. These included the markers *Bcalμ10*, *C203*, *C218*, *D210*, *D106*, *C205*, *C223*, *D105* [29]. Markers were amplified in multiplex PCRs (details in Additional file 1: Table S1) and run on a ABI3130 genetic analyzer. Alleles were scored using Genemapper 4.0 (Applied Biosystems) and genotypes checked for null alleles using micro-checker [30].

### Phylogenetic analyses

Phylogenetic reconstruction of the mitochondrial control region was performed using PhyML [31]. Analyses included a model of HKY + G selected by jModelTest [32] (AIC criteria) and 1′000 bootstrap pseudo-replicates. We estimated the divergence of the main lineages in BEAST [33], using three known calibration points on well-resolved nodes: the divergence between *B. siculus*/*B. boulengeri* and *B. balearicus*/and the lineages *viridis*/*variabilis* (2.6 Mya, 3.3–1.9 Mya), the split between the sister taxa *B. siculus* and *B. boulengeri* (1.8 Mya, 3.5–0.63 Mya) and the split of *B. balearicus* from *B. viridis*/*variabilis* (1.9 My, 2.5–1.3 Mya) [6, 17, 18]. In the absence of appropriate fossils that can be related to certain mtDNA lineages [16] these are the best available calibration points for divergence estimations in Palearctic green toads (cf. [17]). Molecular dating involved a Yule speciation tree and normally distributed priors (with prior distribution spanning the estimated range of divergence times). We ran four independent chains of 10 million iterations each, and visualized parameters in Tracer to ensure convergence and sufficient effective sample size (ESS > 200), as well as the phylogeny in FigTree.

For the slower-evolving nuclear *tropomyosin* intron (*tropo*), the unresolved phylogeny of the lineages present in the study area precluded from sound phylogenetic inferences and molecular dating. We thus analyzed sequence differentiation by means of a haplotype network approach under a 95% parsimony limit (TCS, [34]).

### Population genetic analyses

We explored patterns of population diversity and structure from the microsatellite dataset in several ways. First, we applied the Bayesian clustering algorithm of STRUCTURE [35] testing population clustering from 1 to 11 groups (K), with 100,000 iterations per K (after a “burnin” period of 10,000), which ensured stationarity and convergence. The number of groups best-explaining the data was assessed by two commonly used statistics, computed by STRUCTURE HARVESTER [36]: the average log-likelihood Pr(X│K) [35] and the ΔK [37]. Replicate runs were combined with CLUMPP [38] and graphs of assignment probabilities were compiled using DISTRUCT [39]. We also conducted additional STRUCTURE analyses with K = 2 on the continental genotypes only (loc. 20–106), to infer patterns of introgression at the *viridis* / *variabilis* contact zone. Second, we performed a Principal Component Analyses (PCA) of population allele frequencies on the full dataset, using PCAgen (http://www2.unil.ch/popgen/softwares/pcagen.htm), where significance of axes was tested by 10,000 permutations. Third, we estimated the genetic diversity on each island by computing observed heterozygosity (H_o_), allelic richness (A_r_), inbreeding coefficient (F_is_), and tested for Hardy-Weinberg Equilibrium (HWE) using FSTAT [40]. We also assessed mitochondrial diversity by computing haplotype diversity (H_d_) and nucleotide diversity (п) using Arlequin [41]. Given their admixed genetic nature, we did not perform such analyses for the continental populations.

## Results

### Mitochondrial analyses

**Fig. 1:**
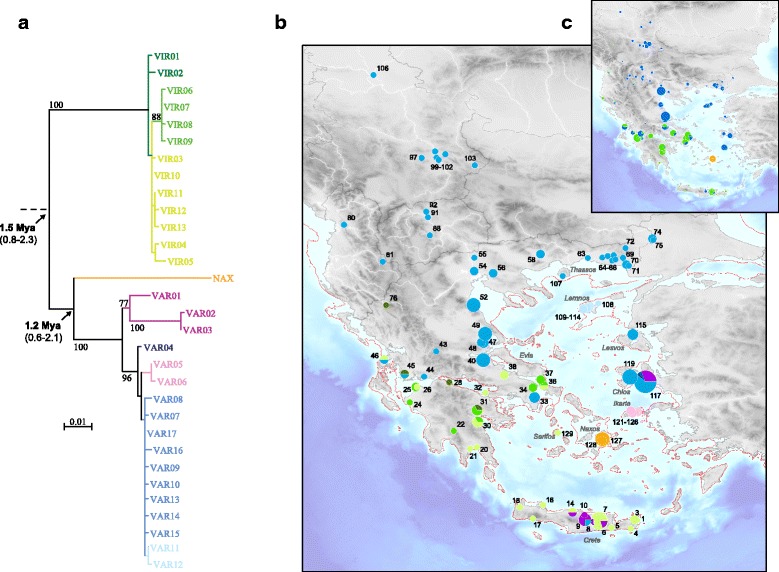
**a** Maximum-likelihood mitochondrial phylogeny, **b** distribution of the main haplogroups of the mitochondrial control region, based on 165 sequenced individuals, and **c** fine-tuned distribution of green toad lineages based on 679 mitotyped samples. For **b**, colors correspond to the haplogroups of the tree; the red line shows the shoreline at the Last Glacial Maximum. For **c**, colors correspond to the mtDNA lineages as follow. Green: *viridis*; blue: *variabilis*; orange: endemic lineage from Naxos. Bootstrap support are shown for major branch (based on 1′000 pseudo-replicates); on the maps, circle sizes are proportional to sample size

In the study area, analysis of an alignment of 862 bp from the mitochondrial control region (115 variable sites, 71 parsimony-informative) revealed 31 haplotypes. These formed three main lineages: (1) the *viridis* clade (13 haplotypes), distributed on Crete, Serifos, the Peloponnese and in Central Greece, with weak differentiation and little geographic association of haplogroups (green clade, Fig. 1). (2) The much more structured *variabilis* clade (17 haplotypes), distributed across Central and Northern Greece, and in Albania, FYR Macedonia, Serbia, W-Anatolia (blue / purple clade, Fig. 1), as well as on the Aegean islands of Lemnos (with private haplotypes VAR11–12, light blue), Lesvos, Chios and Ikaria (with private haplotypes VAR05–06, pink) and in Central Crete. (3) A clade, endemic to the island of Naxos, represented by a single haplotype (orange clade, Fig. 1; NAX), which is deeply diverged from *variabilis*. Our samples from W-Anatolia (loc. 117) feature very diverse *variabilis* haplotypes (VAR01–04, VAR16–17), some of which are elsewhere only found in Central Crete.

Molecular dating estimated the divergence between these clades to the lower Pleistocene, i.e. 1.5 Mya (95% HDP: 0.8–2.3 Mya) for *viridis*, and 1.2 Mya (95% HDP: 0.6–2.1 Mya) for the new Naxos lineage and the lineage of *variabilis*.

Insular diversity of the control region was highly variable between islands (Table 1), exhibiting particularly high values for Crete, where both *variabilis* and *viridis* haplotypes coexist (resulting in high п), and the lowest values for Naxos and Lesvos (each with a single fixed haplotype).

**Table 1:** Diversity estimates in insular green toads from the Aegean

	microsatellites				mitochondrial		
	n	H_o_	A_r_	F_is_	n	H_d_	п
Crete	51	0.49	3.63	−0.09	29	0.48	0.0260
Lemnos	47	0.48	3.69	0.00	11	0.18	0.0002
Chios	11	0.64	4.38	0.05	6	0.53	0.0006
Ikaria	32	0.47	3.60	−0.02	8	0.25	0.0003
Naxos	23	0.23	2.22	−0.10	7	0.00	0.0000
Lesvos	13	0.67	5.31	−0.01	3	0.00	0.0000
Evia	46	0.57	4.69	−0.02	4	0.83	0.0029

H_o_: observed heterozygosity; A_r_: allelic richness; F_is_: inbreeding coefficient (scaled to five individuals); H_d_: haplotype diversity; п: nucleotide diversity; n: sample site

Mitotyping-without-sequencing for the rest of our samples allowed to fine-tune the distribution of the mitochondrial lineages *variabilis* and *viridis*, especially in their contact zone in Greece (Fig. 1c), where both haplotypes are shared by many widespread populations (27–52). While many *viridis* haplotypes dominate the Peloponnese, a single *variabilis* and *viridis* mtDNA appear to co-exist on the western parts of the Balkan Peninsula (loc. 78–80, 84, 95).

### Nuclear sequence analyses

We identified 14 haplotypes of the nuclear *tropomyosin* (alignment: 555 bp), with some geographic associations. The eastern parts of the study area (*variabilis*) featured highly differentiated sequences shared by individuals from across W-Anatolia, Chios, Lesvos and Ikaria (green, light blue, blue, dark blue, black, pink, Additional file 2: Figure S1). As for mtDNA, Naxos green toads possessed a single *tropomyosin* haplotype (orange, Additional file 2: Figure S1). Individuals from southern Greece harbored haplotypes closely-related to a previously published *B. viridis* sequence (Genbank EU497619). However these haplotypes were also found in the eastern Aegean (loc. 117, 119), probably resulting from incomplete lineage sorting with the polymorphic *variabilis* lineage (see Discussion).

### Population genetics analyses

**Fig. 2:**
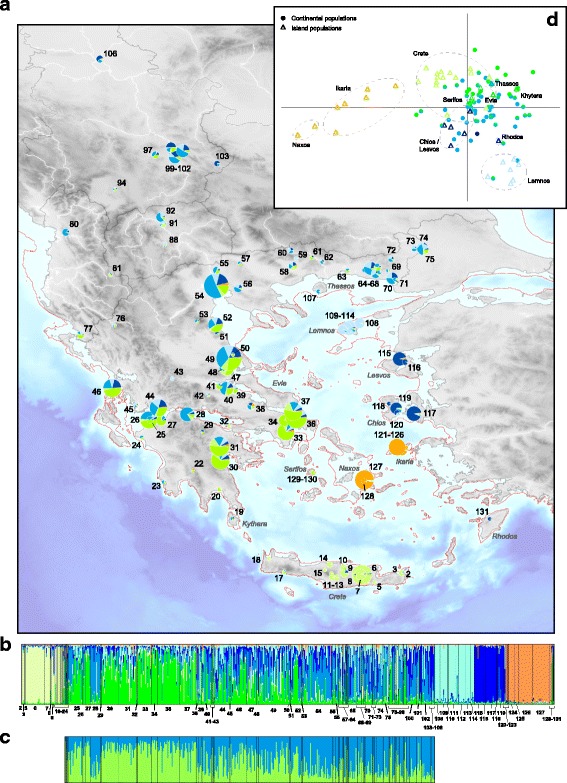
Individual based assignments of nuclear genotypes by STRUCTURE and population-based PCA. **a** Average admixture coefficient among populations, for K = 6; circle sizes are proportional to sample sites; the red line shows the shoreline at the Last Glacial Maximum; **b** individual assignment with K = 6; **c** Individual assignments of continental green toads with K = 2, distinguishing the lineages of *viridis* (green) and *variabilis* (blue); **d** First two axes of the population PCA. Triangles and circles discriminate between insular and continental localities, respectively Major islands are encircled. Colors correspond to the clustering of STRUCTURE

We did not detect null allele or scoring errors in our microsatellite dataset. Bayesian assignment of microsatellite genotypes showed consistent geographic structure as inferred by mtDNA (Fig. 2a-b). The ΔK statistics indicated a conservative solution of K = 2, discriminating populations from Naxos and Ikaria from the rest of the range, with intermediate assignments in Crete (Additional file 3: Figure S2). The Pr(X│K) statistic did not improve beyond K = 6 (Additional file 3: Figure S2), with the following geographically associated clusters: (i) Naxos and Ikaria (orange); (ii) *variabilis* from W-Anatolia, on Chios and Lesvos (dark blue); (iii) *variabilis* from Lemnos (light blue), (iv) European *variabilis* (blue), (v) *viridis* from Crete (light green), and (vi) continental *viridis* (green). As for mtDNA, European *variabilis* (iv) and continental *viridis* (vi) widely admix in central Greece, featuring intermediate admixture proportions (loc. 25–54). Hierarchical STRUCTURE analyses from K = 2 to K = 6 show that the Cretan and continental *viridis* cluster together at low K values; similarly, the analyses progressively differentiated the three *variabilis* clusters as K increases; however, for K = 3, the eastern populations were partially associated with the Cretan ones (Additional file 3: Figure S2). Additional analyses (based solely on continental populations), also suggested strong admixture across the study area between the lineages *viridis* and *variabilis* (Fig. 2c). The population PCA provided a similar signal. The first axis (23% of variance explained) differentiated Naxos, Ikaria and all remaining populations (Fig. 2d). The second axis (14% of variance explained) separated the lineages *variabilis* and *viridis* from each other, with the insular populations (Crete, Lemnos) of both lineages showing profound deviations from those on the mainland. Populations from the contact zone in central Greece strongly overlap in the PCA output. Interestingly, the six populations sampled on Ikaria exhibited highly variable scores on the first axis, intermediate between those of Naxos and *viridis*/*variabilis*.

Insular diversity based on microsatellites closely matched estimates obtained from mtDNA, with some variation between islands (Table 1). In fact, considering only islands with meaningful sample sizes (*n* ≥ 5), microsatellite H_o_ and A_r_ correlates with mitochondrial haplotype diversity (Pearson’s correlation, *r* = 0.88 and 0.85 respectively), although the small number of comparable populations (*n* = 5) prevents reaching statistical significance (*P* = 0.05 and 0.07, respectively). No insular population departed from HWE, with F_is_ closed to 0.0 (Table 1).

## Discussion

Although the Aegean islands and coasts are bearing terrestrial phylogeographic signatures from the Mid-Aegean Trench (12–9 Mya), the Messinian Salinity Crisis (5.3 Mya), Pleistocene sea level oscillations and Holocene human impacts [20, 22], the colonization of green toads appears mostly or entirely determined by (Plio-) Pleistocene and younger processes. Several patterns of genetic differentiation in Aegean green toads correlate well with the paleo-fluctuations of the sea levels during Pleistocene glacial cycles. At the LGM, the Aegean Sea was ~ 120 m lower than today [42, 43], remodeling the coastline deep below the current one (red in Figs. 1 and 2). The central Cyclades have remained completely isolated, which promoted the secluded evolution of green toads on Naxos throughout the Pleistocene. In contrast, most eastern islands (Lesvos, Chios, Rhodes) were either part of the mainland, or accessible by narrow landbridges (Ikaria) during the LGM and/or supposedly during previous glacial periods, thus accounting for the weak divergence of these populations. The genetic differentiation on Ikaria and Lemnos may either suggest rapid isolation after the LGM or imply that the latest connections date back to older glacial periods. The following sections detail these main findings, and discuss the genetic interactions between the debated *viridis* and *variabilis* lineages and their evolutionary consequences.

### A new endemic green toad lineage on Naxos

We discovered a new green toad lineage, endemic to the island of Naxos in the central Cyclades, supported by concordant mitochondrial (deeply-diverged mtDNA clade, Fig. 1) and nuclear signals (private *tropo* haplotype and disruptive microsatellite clustering; Fig. 2 and Additional file 2: Figure S1). This finding of a new deeply diverged mtDNA lineage in European green toads is by itself remarkable. In contrast with the other mtDNA-lineages found across the Aegean islands (*viridis* and *variabilis*), which are widespread throughout northern Eurasia [16, 17], the new lineage is, as far as we know, endemic to a single Aegean island (Naxos). During lowest Pleistocene sea levels, substantial parts of the now submerged Cycladic Plateau were exposed, forming clusters of larger islands or, at periods of extensive sea-level drop, a single mega-island [44]. However, despite intense efforts in spring 2015, we have been unable to find green toads on the episodically connected neighboring island of Paros. The populations on Naxos appear to have remained isolated at least since the Lower Pleistocene (~ 1.2 Mya) and, as the low levels of nuclear and mitochondrial diversity testify, probably experienced historically small effective sizes. With up to 40% endemism, Naxos ranks among the Aegean islands with a high species diversity for amphibian and reptiles [45], and is a well-known endemism hotspot for invertebrates [46]. In addition, an extinct endemic Pleistocene mammal fauna, including dwarf elephants and rock mice, also existed on Naxos and probably other central Aegean islands [44].

In *Podarcis* lizards, endemic lineages occur on Milos and Skyros, two islands in the central Aegean region [47]. Similarly, populations of the snake *Vipera ammodytes* on the Cyclades islands correspond to a unique clade of Pliocene age [48].

### Insular diversification of green toads on other Aegean islands

In contrast to the endemic situation on Naxos, the insular green toad populations along the Anatolian and northern Greek coasts feature weak or no divergence compared to the nearby continents. Yet, sharp genetic structuring and private mtDNA haplotypes strongly suggest the absence of contemporary gene flow, neither with Ikaria nor Lemnos. Islands closer to the mainland (< 10 km and only separated by shallow coastal waters), however, are not genetically distinct, indicative of recent dispersal events. This comprises Lesbos, Chios and Thassos (inhabited by *variabilis*) as well as Evia (inhabited by *viridis* and *variabilis*). Crete makes a special case: while its toads have been assigned to a discrete nuclear cluster, most of the island features *viridis* mtDNA, as previously shown by [16], whereas some central Crete populations feature mitotypes typical of the Anatolian *variabilis* lineage. While the nuclear *tropo* marker illustrates well the greater diversity within *variabilis*, inference from this locus has to be taken with caution given the apparent incomplete lineage sorting of some ancestral haplotypes with its sister species *viridis* (e.g. Chios and Turkey, Additional file 2: Figure S1).

One of the most comprehensively studied systems in this region remains the *Pelophylax* (previously *Rana*) water frogs [25], where endemic species occur in Crete (*P. cretensis*), which was disconnected since the end of the Messinian Salinity Crisis (5.3 Mya), as well as on Karpathos and Rhodes Island (*P. cerigensis*), that were also disconnected throughout the Pleistocene, while populations from coastal Anatolian (e.g. Ikaria, Samos, inhabited by *P.* cf. *bedriagae*) and Greek islands (e.g. Evia, Kythera, inhabited by *P. ridibundus*) are weekly differentiated from the continent. Modern mtDNA analyses have partly reinforced and detailed these early phylogeographic data [49, 50], but found *P. cerigensis* mtDNA to also occur on the Turkish coastal mainland [51].

However, the role of Quaternary climatic oscillations for the Mediterranean sea levels does not reconcile some of the puzzling exceptions in the phylogeographic patterns found among Aegean green toads. On Serifos (*viridis*), Kythira (*viridis* / *variabilis*) and Rhodes (*variabilis*), the few toads analyzed there cluster with mainland ones, although the islands are separated by deeper sea floor (> − 200 m) and supposedly disconnected even during the driest Quaternary periods [24]. The short distance to adjacent coastlines could have allowed stepping-stone transmarine dispersal. The Aegean region is tectonically active and uplifts can rapidly occur, temporarily creating landbridges during dry periods [52, 53]. Total isolation of some of these islands thus remains in part uncertain [25].

**Fig. 3:**
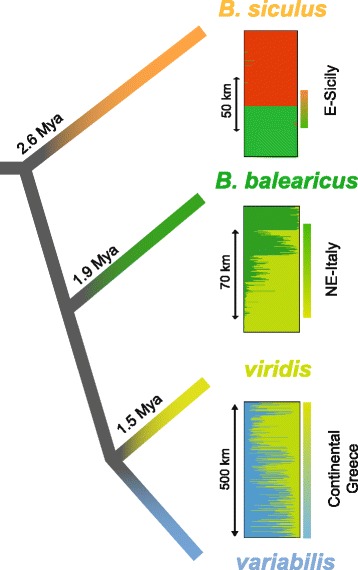
Patterns of introgression in secondary contact zones between incipient species of Palearctic green toads. Adapted from [6, 18], and this study. Barplots show nuclear individual assignments. Gradient rectangles show the distribution of mtDNA. Indication of distances at localities from the contact zones are indicated, as well as divergence times of lineages, estimated from mtDNA

The discrepancies between mtDNA and nuclear signals on the island of Ikaria are also worth discussing. These populations feature *variabilis* mitotypes despite being nuclearly more similar to the endemic Naxos lineage, however with intermediate scores on the main PCA axes (Fig. 3). One explanation could be that the Naxos lineage used to also extend to Ikaria (the two islands are separated by less than 80 km), which later got invaded by mainland toads via a former land bridge. The ensuing hybridization would then have fixed *B. variabilis* haplotypes and admix the nuclear gene pool of Ikarian toads. Additional samples from neighboring islands and coastal areas would help testing this scenario and clarify the distribution of lineages in this relatively understudied part of the Western Palearctic.

### Potential influence of humans on insular Aegean green toads

Potential translocations by human settlers provide a plausible explanation for some unusual patterns. Indeed, human-mediated introductions may have largely contributed to the colonization of Crete from multiple sources, most likely during antique times. While Crete has been geologically disconnected from the mainland for at least 5.3 million years, Cretan green toads, which are widely distributed over the island, mostly share *viridis* mitotypes of Peloponnese origin [16]. The clear-cut nuclear differentiation compared to the mainland, however, argues for an absence of contemporary colonization, thus pointing to historical translocations. The Aegean region has been inhabited since the early Pleistocene by Neanderthals and modern humans [54]. Crete was then home of the Minoan civilization, one of the most ancient in Europe (2700–1420 BC), before being overrun by the Mycenaeans from mainland Greece (1420–1375 BC). Green toads could have been imported from the Peloponnese during both eras, via trading of the Minoans with the Greeks, and/or later during the Greek invasions, which were subsequently followed by massive waves of immigration during the Archaic Period (800–500 BC) [20]. Moreover, the occurrence of Anatolian *variabilis* mitotypes restricted to the vicinity of Heraklion, points to additional eastern contacts post-dating the *viridis* colonization. Since the Byzantine periods, during which exchanges with Asia Minor reached their climax, Heraklion has been an important trading post of Crete. Our results thus emphasize potential effects of ancient Aegean civilizations on species assemblages. Trade routes from Asia and mainland Greece also promoted the colonization of Crete by other vertebrates including several species of amphibians and reptiles, like the snake *Zamenis situla* [55], the geckos *Tarentola mauritanica* from Tunisa [56] and *Hemidactylus turcicus* from the Middle East [57], the skinks *Chalcides ocellatus* from Libya [58] and *Ablepharus kitaibelli* probably from Karpathos [59], the tree frog *Hyla arborea* from the Peloponnese [60], and the shrew *Crocidura suaveolens* from Anatolia [61].

### Patterns of admixture between *viridis* and *variabilis* on the Greek mainland

We show that the two lineages *viridis* and *variabilis* form a hybrid swarm across central Greece. In our study area, *viridis* is restricted to the Peloponnese and SE-Greece. On the Greek mainland, a single *variabilis* haplotype (VAR17), widespread across the southern Balkans (Fig. 1), presumably indicates a recent invasion of this eastern lineage expanding westwards within the genetically richer *viridis* refugium, which is composed of several mtDNA haplotypes. In the study area, while *viridis* mitotypes do not reach further northeast than Thessaly (but otherwise widespread in Western and Central Europe), nuclear introgression was found as north as Macedonia. It is unclear, whether the weak signs of *viridis* admixture across the Thrace province, here picked up by STRUCTURE (Fig. 2), reflect true introgression, or rather “background noise” due to a higher nuclear diversity of *variabilis* closer to its Black Sea and Anatolian refugia [14]. Given the intermediate admixture coefficients on the mainland, with no seemingly “pure” individuals (Fig. 2b), it is possible that both toad lineages, despite the clear evidence of a secondary contact from the two mtDNA clades (*viridis*, *variabilis*), now admix to a point of near-panmixia, and that little or no actual genetic structure remains. The Balkans, where scarce samples also appear admixed, may host another wide hybrid zone between both lineages. No noticeable patterns of cyto-nuclear discordances were observed: the average admixture proportions at nuclear loci parallel the relative frequencies of mitotypes in these populations (Figs. 1 and 2).

### On the evolution of reproductive isolation in diploid European green toads

The other major aim of our study was to compare the patterns of hybridization between the Eurasian mitochondrial sister lineages *viridis* and *variabilis* with those in other diploid/diploid green toad contact zones of older divergence. As discussed by [19], the taxonomic status of the mtDNA lineages *viridis* and *variabilis*, whose distribution also clusters geographically [16, 17, 19], has remained unclarified. As we show here, no major (if any) reproductive barriers seems to separate the two gene pools. The focal region exhibits massive mitochondrial and nuclear admixture over hundreds of kilometers. This wide admixture supports that *viridis* × *variabilis* natural hybrids are not selected against, suggesting poor or complete absence of prezygotic and probably no post-zygotic incompatibilities between these two lineages.

This observation is well in line with the prediction that hybridizability (or introgression, respectively) correlates with the amount of allopatric divergence, a hypothesis with accumulating evidence from several vertebrate groups [3, 4, 6, 10]. The Plio-Pleistocene diverged *B. siculus* and *B. balearicus* (2.6 Mya) are virtually non-admixing at their Sicilian secondary contact [18], while the latter forms a ~ 50–100 km wide hybrid zone with the Lower Pleistocene-diverged *B. viridis* in N-Italy (1.9 Mya; [6]). Here we presented a third point for assessing introgression in green toads under natural conditions: *variabilis* and *viridis*, diverged in the Mid-Pleistocene (1.5 Mya), widely admix over hundreds of kilometers across the Balkan Peninsula (Fig. 3). Therefore, it would not be justified to maintain the name *B. variabilis* (Pallas, 1769) in the status of a separate species under the conservative biological species concept [62]. This taxon may rather be considered a subspecies epitheton, i.e. with *B. viridis viridis* and *B. viridis variabilis*). However, it remains unclear whether these lineages similarly admix in other parts of their Eurasian range, where they feature different patterns of diversity and distribution. In another bufonid hybrid system, *Bufo bufo* / *B. spinosus*, comparative hybrid zone analyses revealed contrasting amounts of admixture across several replicate contact zones, differing in terms of genetic variation and the mitochondrial lineages involved [63].

In conclusion, reproductive isolation seems to have accumulated gradually in green toads and may mainly stem from the additive effects of weak hybrid incompatibilities spread over the genome, rather than few important speciation genes. The evolution of reproductive isolation involves multiple interacting barriers in a continuous framework [1]. In the Palearctic green toad radiation, different stages along this continuum have evolved, which thus provides unique insights into the timeframe when gene flow might either promote complete isolation (through the evolution of pre-mating barriers, i.e. reinforcement) or (re-)merge gene pools. In the latter case, while gene flow cancels the intrinsic identity of incipient species, it enables new genetic combinations and thus increases the adaptive potential of populations, which may in turn promote future speciation processes (e.g. ecological speciation). In green toads, this temporal window seems quite narrow: only a million years separates a scenario of near panmixia (lineages of *viridis* / *variabilis*, this study) from a situation of complete reproductive isolation, potentially driven by reinforcement (*B. siculus* / *B. balearicus*; [18]). Between these extremes, the *B. balearicus* / *B. viridis* contact provides a presumably intermediate stage [6], with preliminary indications for ecological and / or behavioral adaptations keeping lineages apart (our unpublished data). Beyond intrinsic genetic causes, environmental and demographic features could affect this timeframe. Reinforcement can only drive divergence if secondary contacts are prolonged enough to allow a selective response to post-zygotic isolation. In Europe, such contacts primarily include the Mediterranean regions, where populations persisted during the Quaternary glaciations, rather than post-glacially colonized more northern latitudes, where secondary contacts could only be initiated since the Holocene. Nevertheless, this should not have affected our comparisons as shown in Fig. 3, since all population pairs were analyzed in supposedly refugial areas. Moreover, all three analyses ([6, 18], this study) were also highly comparable given that the same mitochondrial and nuclear markers have been used, both for estimating (relative) divergence and natural introgression.

Similar conclusions have been drawn from two other hybrid zone analyses in *Hyla* tree frogs [7], where incompatibilities appear to disproportionally accumulate on sex chromosomes [64]. However, compared to green toads, the time taken to speciate could be twice longer in *Hyla* (up to ~ 5 My), suggesting slower accumulation of incompatibilities and/or less opportunities for the evolution of pre-zygotic barriers [7]. Such sharp differences may also depend on ecological context and/or life-history traits, such as dispersal rates and prepotency for local adaption [65, 66], which could add ecological components to speciation processes (cf. [67]). Genetic mapping of hybrid incompatibilities to infer their underlying causes would shed some light, a method particularly promising with hybrid zone analyses [1]. In any case, it stresses for similar analyses in additional anuran radiations in order to draw meaningful quantitative inferences on the time taken to speciate in allopatry.

## Conclusions

Our multilocus phylogeographic analyses of Aegean green toads allowed to test biogeographic and speciation hypotheses with several new findings. First, we detected a new cryptic endemic lineage in the central Aegean (Naxos), along with strong population structure between other islands, mediated by the depth-dependent Pleistocene sea level dynamics around the Archipelago, and presumably involving human-driven colonization(s) during antique times. Second, we show that the young lineages *viridis* and *variabilis* massively admix across their secondary contact zone in mainland Greece, presumably driven by an eastern invasion of *variabilis*, contrasting with the patterns of introgression between more deeply diverged green toad lineages in other range parts. In green toads, post-zygotic reproductive isolation appears mediated by the time spent in allopatry, upon which gene flow either promotes or cancels the speciation process. This radiation thus provides an increasingly valuable anuran model system for speciation research (including allopolyploids in Asia, [68]), as it illustrates different time spans of secondary contacts along the speciation continuum.

## Additional files

Additional file 1: **Table S1.** Information on the localities sampled in this study (A) and details on the microsatellite multiplexes (B). (XLSX 20 kb)

Additional file 2: **Figure S1.** Haplotype network of the *tropo* intron marker, and distribution of the main haplogroups. Circles haplotypes show reference sequences for each species. Colors were tentatively attributed to the main nuclear clusters inferred from microsatellites. (PDF 4837 kb)

Additional file 3: **Figure S2.** (A) Hierarchical STRUCTURE analyses from K = 2 to K = 6 on the entire dataset; (B) Pr(X│K) and ΔK statistics computed by STRUCTURE HARVESTER from the analyses on the full dataset and on continental populations only. (PDF 1016 kb)
