# Supplementary material for: Phylogeography of Aegean green toads (Bufo viridis subgroup): continental hybrid swarm vs. insular diversification with discovery of a new island endemic
Source: BMC Evol Biol. 2018 May 2;18:67. doi: 10.1186/s12862-018-1179-0 (PMC5930823; doi:10.1186/s12862-018-1179-0)
Supplement: Supplementary file 2 — Figure S1. Haplotype network of the tropo intron marker, and distribution of the main haplogroups. Circles haplotypes show reference sequences for each species. Colors were tentatively attributed to the main nuclear clusters inferred from microsatellites. (PDF 4837 kb) [file 12862_2018_1179_MOESM2_ESM.pdf]

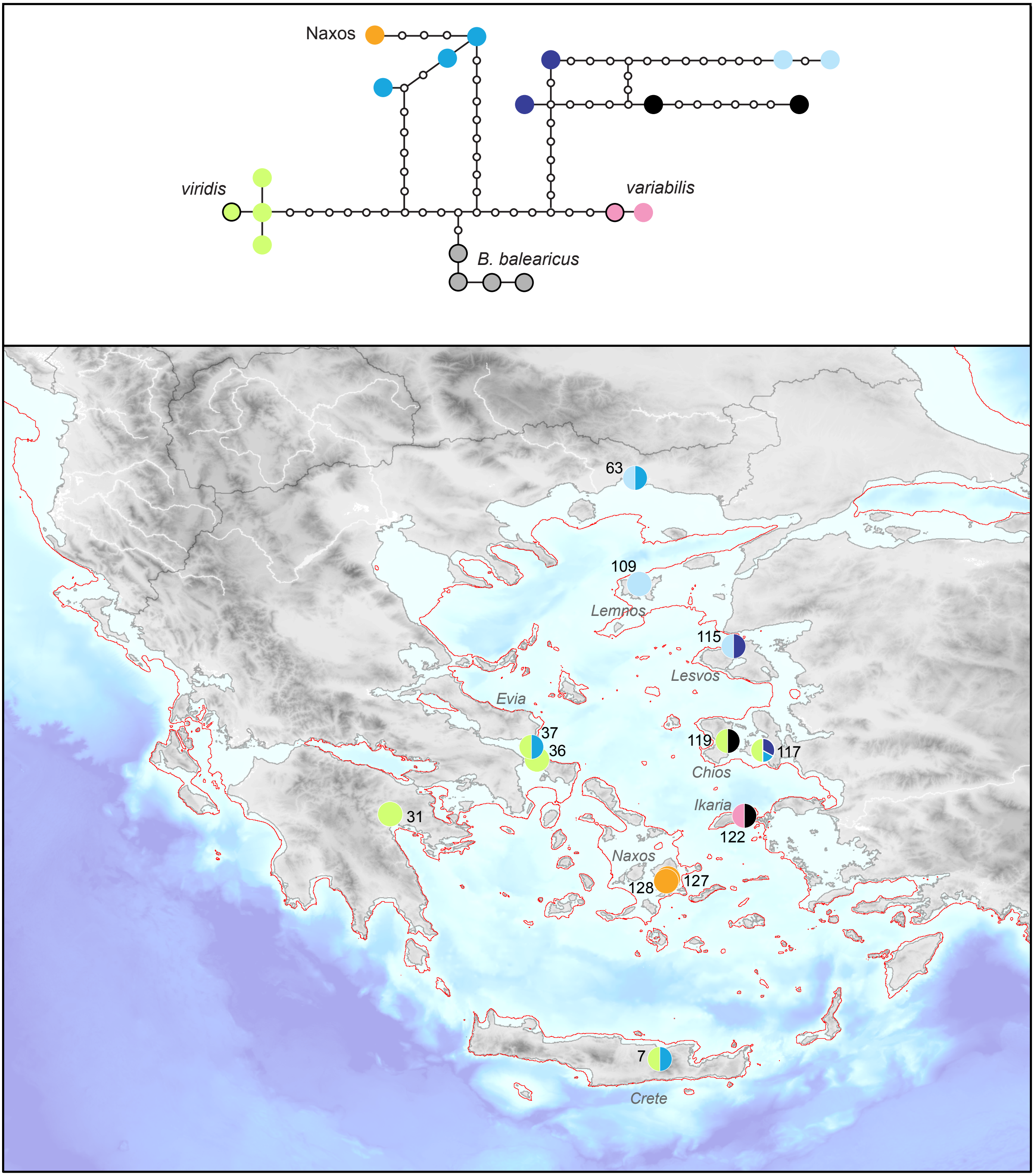

Fig. S1: Haplotype network of the *tropo* intron marker, and distribution of the main haplogroups. Circles haplotypes show reference sequences for each species. Colors were tentatively attributed to the main nuclear clusters inferred from microsatellites.
