# Supplementary material for: Phylogeography of Aegean green toads (Bufo viridis subgroup): continental hybrid swarm vs. insular diversification with discovery of a new island endemic
Source: BMC Evol Biol. 2018 May 2;18:67. doi: 10.1186/s12862-018-1179-0 (PMC5930823; doi:10.1186/s12862-018-1179-0)
Supplement: Supplementary file 3 — Figure S2. (A) Hierarchical STRUCTURE analyses from K = 2 to K = 6 on the entire dataset; (B) Pr(X│K) and ΔK statistics computed by STRUCTURE HARVESTER from the analyses on the full dataset and on continental populations only. (PDF 1016 kb) [file 12862_2018_1179_MOESM3_ESM.pdf]

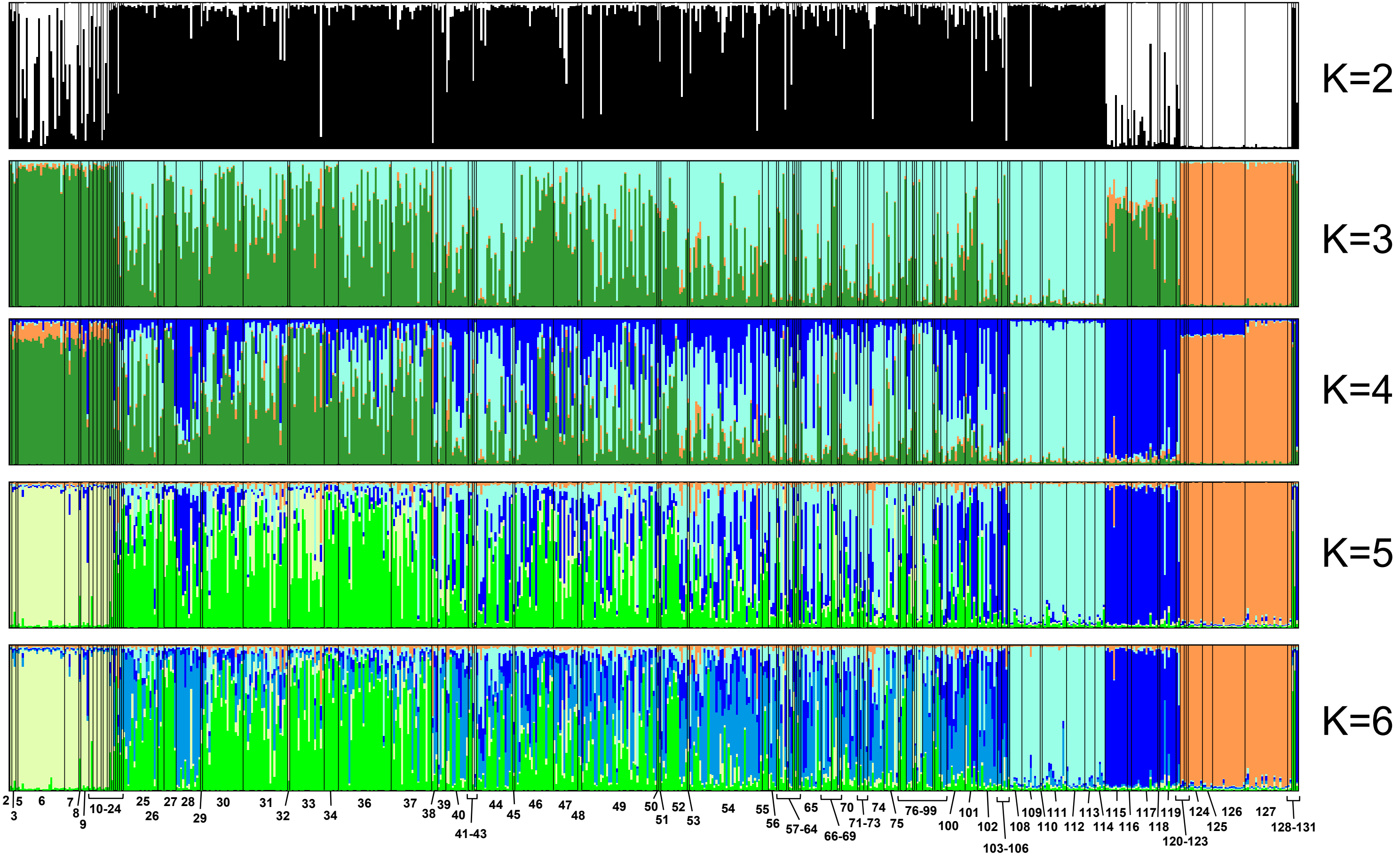

Fig. S2A: Hierarchical STRUCTURE analyses from K = 2 to K = 6.

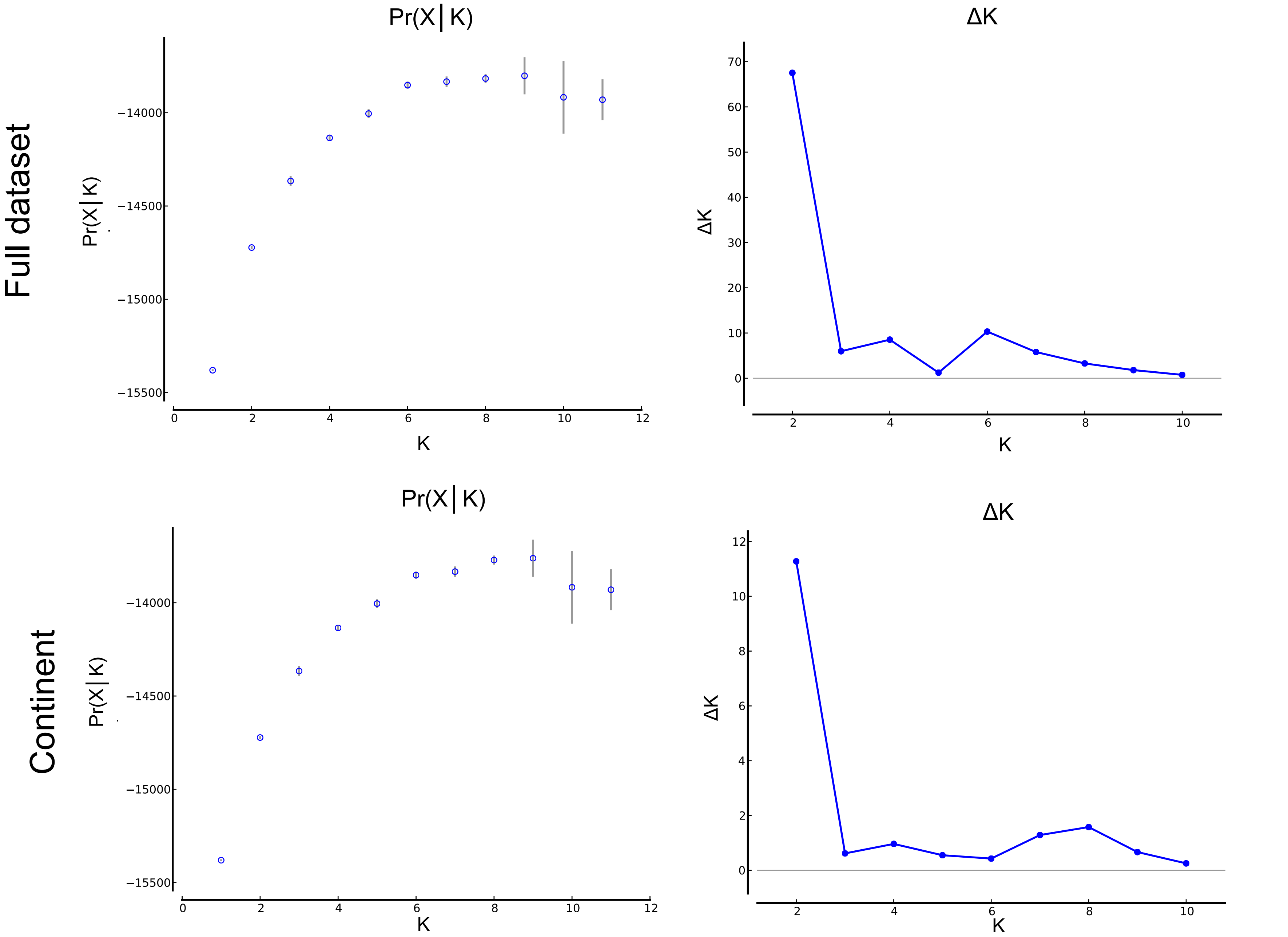

Figure S2B: Pr(X|K) and ΔK statistics from the STRUCTURE analyses of microsatellites genotypes based on the entire dataset or on continental populations only
